# Community engagement to support public health: mixed-method evaluation evidence on COVID-19 attitudes and practices in Lao PDR

**DOI:** 10.1080/16549716.2025.2485523

**Published:** 2025-04-25

**Authors:** Marco J. Haenssgen, Elizabeth M. Elliott, Sandra Bode, Ounkham Souksavanh, Thongkhoon Xayyahong, Hironori Okabayashi, Shogo Kubota

**Affiliations:** a Department of Social Science and Development, Chiang Mai University, Chiang Mai, Thailand; b Maternal Child Health and Quality Safety, World Health Organization Regional Office for the Western Pacific, Manila, Philippines; c World Health Organization Representative, Country Office for Lao People’s Democratic Republic, Lao PDR

**Keywords:** Community engagement, evaluation, COVID, antimicrobial resistance, rural areas, Lao PDR

## Abstract

**Background:**

Community engagement has been recognized as a key tool for supporting national health agendas, and experiences from the COVID-19 pandemic can offer important lessons for tackling future global health challenges such as antimicrobial resistance. This paper provides much-needed evaluation knowledge on relational community engagement initiatives and their impact on COVID-19-related attitudes and practices.

**Methods:**

A two-round mixed-method evaluative study to examine outcome indicators related to COVID-19-prevention and health-seeking behavior was implemented from October 2022 to December 2023 among 14 diverse case study communities in four Lao provinces. Data involved 50 semi-structured interviews with villagers, 50 key informant interviews, and two rounds of complete census surveys (3,161 survey observations incl. matched panel data from 618 individuals) to discern outcomes among villagers with different levels of activity participation in a difference-in-difference analysis.

**Results:**

Relative to non-participating villagers, villagers participating in the activities had higher COVID-19 vaccine uptake (+0.13 doses), higher public healthcare utilization for presentations consistent with COVID-19 (e.g. fever and neurological and/or respiratory symptoms; +69.4% points), and less antibiotic use per illness episode (−0.2 antibiotic use episodes). However, the activity raised worries to disclose a COVID-19-positive status and was often interpreted as a health education campaign.

**Conclusions:**

Relational community engagement offers a respectful way of addressing persistent healthcare challenges and supporting vulnerable populations – and thus holds key for ongoing global health priorities such as emerging infectious disease responses and antimicrobial resistance. We recommend that community engagement initiatives become a standard component of national health policy portfolios beyond the scope of COVID-19.

## PAPER CONTEXT

- **Main findings**: A nationwide community engagement initiative in Lao PDR achieved intended improvements in COVID-19-related attitudes and practices and unintended improvements in judicious antibiotic use – alongside minor yet rectifiable undesirable outcomes.
- **Added knowledge**: This study provides important short-term evaluation knowledge on the viability and positive potential of relational community engagement approaches that emphasize trust between communities and the health system.
- **Global health impact for policy and action**: Community engagement has received increasing attention during the COVID-19 pandemic and our evaluation underlines its important role for the global and national health policy toolkit in the post-pandemic era.

## Background

### Introduction

Community engagement – generally understood as both a process and outcome of community-led multi-stakeholder cooperation to address local health-related issues [1,2] – holds formidable potential for the continued advancement of healthcare service delivery, organization, and ownership services. While the interpretation of the concept varies, it has a long legacy in global health practice reaching back to the growth of participatory development in the 1980s and the Alma Ata primary care movement 1978 [3,4]. Its latest evolution of ‘relational’ approaches to community engagement revolves around fostering relationships and foregrounding the agency of target populations without pushing external knowledge and solutions on them [3,5]. Such an approach holds promise to revolutionize the relationship between people and the health system toward fundamentally more trusting, understanding, and respectful interactions. The potential of community engagement had accordingly led it to gain widespread recognition in global health policy, both during and before the COVID-19 pandemic [6,7].

Owing to the syndemic character of the COVID-19 pandemic with social roots in intersectional disadvantage that aggravated parallel global health priorities such as antimicrobial resistance [8–10], community engagement lessons learned (and their particular relevance for vulnerable populations) are key to tackle future global health challenges and recurring infectious disease outbreaks [11]. However, the overall impact evidence of community engagement activities during the COVID-19 pandemic remains limited and focused primarily on instrumental rather than relational approaches.

The objective of this article is to contribute to the understanding of the opportunities of community engagement initiatives in supporting public health objectives, such as emerging infectious disease responses with specific reference to COVID-19 experiences. The research took place across rural Lao PDR and in conjunction with a nationwide initiative to strengthen primary healthcare services that the Lao Ministry of Health (MoH) and Ministry of Home Affairs (MoHA) implemented with technical support from the World Health Organization (WHO). This initiative was borne out of healthcare challenges experienced during the COVID-19 pandemic and involved support for localized healthcare governance and a multi-sectoral approach to empower communities to jointly improve the health of the people in Lao PDR [12]. Relationship building and healthcare ownership through trust-building engagement activities on the village level played a central role in this process; the initiative became accordingly referred to as Community Network Engagement for Essential Healthcare and COVID-19 Responses Through Trust (CONNECT).

Lao PDR’s COVID-19 experience appeared relatively less severe than other nations, as it was the last country in Southeast Asia to register its first positive case in March 2020 and the first COVID-19-related death as late as May 2021 [13,14]. However, challenging national and geopolitical circumstances during and after the pandemic have disrupted health service provision, public finances, and macroeconomic stability in Lao PDR. The country responded to these stressors, among others, with initiatives to increase the efficiency of existing health programs and to re-prioritize primary healthcare services [15–18]. This underlines the relevance of CONNECT as an innovative means to support future health policy in Lao PDR.

### The CONNECT initiative

The CONNECT initiative was designed to empower citizens and local officials to seek health solutions that are realized together for their community [12]. The initiative aimed to collaborate with local officials through the Lao Ministry of Health (MoH) and Ministry of Home Affairs (MoHA) to prepare for, prevent, and respond to the potential widespread transmission of COVID-19 and enhance community health beyond COVID-19 to achieve Universal Health Coverage and health-related Sustainable Development Goals.

The CONNECT initiative was thus motivated by the political attention to health and the challenges created by the COVID-19 pandemic alongside persistently high maternal and child mortality. Unlike technical health promotion initiatives, however, CONNECT was rooted in the understanding that an effective response would require systemic change toward multi-sectoral coordination and local governance of primary healthcare as well as the need for building good relationships and trust through an adaptive and bottom-up approach centred on communities themselves (the development process is described in detail in Kubota et al. [19]). CONNECT was thus developed for MoH and MoHA to jointly orchestrate a multi-sectoral coordinated approach through strengthening local governance to leverage community resources to collectively respond to health issues particular to that community.

The roll-out of CONNECT commenced in December 2021 (and was still ongoing at the time of manuscript submission), targeting high priority ‘focus’ districts and villages nation-wide. It covered all provinces and Vientiane Capital and comprised three modules with the overarching aim of boosting health equity in Lao PDR (see Table 1). The three modules were part of a broader process of systemic change toward the local governance of primary healthcare (e.g. by reshaping multi-sectoral collaboration on the provincial level or helping communities define health priorities and action plans) and together aimed to achieve a set of five interlinked operational objectives, namely:

- *Objective 3.1*: Strengthened central/local health governance
- *Objective 3.2*: Enhanced community engagement
- *Objective 3.3*: Enhanced trust
- *Objective 3.4*: Increased service utilization
- *Objective 3.5*: Improved healthcare service quality

**Table 1.** Overview of CONNECT initiative activities.

	Module 1	Module 2 (*focal module in this study*)	Module 3
Focus	Strengthened local health governance in alignment with *Samsang* to respond to COVID-19 and primary healthcare challenges	Empowered and resilient communities to collectively and effectively respond to COVID-19 and primary healthcare challenges in their community	Strengthened health professionals’ capacities to provide COVID-19 and essential maternal and child health services through a respectful and people-centered approach
Target	Health service governance: administrative and health authorities from village, district, and provincial levels	(Mainly) rural communities and frontline public health service providers: village representatives, villagers (esp. ‘vulnerable’ members incl. pregnant women and women in child-bearing age), and health center staff	Frontline public health service providers: health center staff
Activities	2-day interactive health governance strengthening planning workshop Funding mobilization for district- and province-wide CONNECT implementation	**Workshop**: 2-day facilitator training; 2-day participatory planning workshop with community members and health staff to identify existing resources, build relationships, develop plans; 1-day lessons learned meeting with facilitators **Supportive supervision**: Central & provincial teams support district officials and health centre staff to support village representatives and community members to implement community action plans, as well as scaling up activities and good practices to other villages until these become integrated into routine mechanisms.	3-day healthcare staff training on people-centered care for the safe provision of essential MCH services during COVID-19
Topics	Locally led, government-funded scalability, universal (primary) healthcare access, COVID-19, maternal healthcare services	Ownership of and agency in health service delivery, healthcare access, relationship between healthcare providers and communities, COVID-19, essential healthcare services	Maternal and child healthcare services, COVID-19, counselling skills, respectful care, referral care
Objectives	3.1: Strengthened central/local health governance	3.2: Enhanced community engagement 3.3: Enhanced trust 3.4: Increased service utilization	3.5: Improved healthcare service quality

**Source**: CONNECT Concept Note, Kubota et al. [19].

Of particular interest for the current study is Objective 3.4 – which was partly concerned with COVID-19-related attitudes and practices (alongside maternal and child health and equitable healthcare service utilization) – and the village-based Module 2 activities contributing to its achievement on the community level. Preliminary insights from the ongoing implementation of the CONNECT activities had thereby already indicated the potential to positively influence the relationships between rural communities and their local healthcare providers [12], and that the relational approach championed by CONNECT contributed to an increased uptake of COVID-19 vaccination during a dedicated engagement campaign in Xiangkhouang province in 2022 [20]. This study is a part of a series of evaluations to assess its impacts on COVID-19 attitudes and practices on the micro-level.

### Community engagement initiatives in public health

Community engagement activities such as the CONNECT initiative represent an important conceptual advancement in the delivery, organization, and ownership of healthcare services, which has led them to gain rapid recognition in global health policy even before the pandemic [6,7]. The literature recognized early on that community engagement in general is crucial for successful pandemic responses [5,7,21]. Such activities focus on involving target populations in various aspects of healthcare decision-making, delivery, and communication, while forging and fostering relationships among them and various stakeholders, tailoring action to local contexts, and leveraging participatory tools such as consultations or applied theatre plays [7,21–23].

However, while early (pre-COVID-19) forms of community engagement have been instrumentalized in a top-down fashion to impart desirable knowledge and to mobilize communities for externally defined agendas [21,24], the emphasis on ‘relational’ aspects of healthcare embedded in CONNECT is a recent evolution of the field. This is not to say that notions of relationality and engagement in public health are novel. Community engagement in public health has a long history, tracing back at least to the 1978 Alma Ata conference, which emphasized the importance of participation in primary healthcare [3,4]. Although the sentiment surrounding genuine participation and relationality is widely shared [see e.g. [25], practical expressions of community engagement initiatives in public health have tended to focus on ‘mobilization’ of communities for externally defined objectives using new and ‘engaging’ methods of interacting with them [26–32] – that is, the practice of community engagement has been instrumental rather than empowering. The advancement of the field therefore lies in the practice of a government-mandated initiative to enable communities to define their own health needs, foster trust, and mutual understanding between different parties (see e.g. [33,34] for the practical operationalization and effects of trust as a key effect mechanism of CONNECT), and to gain greater ownership over their health matters. Although CONNECT defines the outcomes of its activities, its setup as an approach corresponds to the sentiment that, ‘Rather than focusing solely on specific projects or outcomes, relational community engagement places a strong emphasis on [first and foremost] developing and maintaining long-term connections and trust within communities’ [5, our emphasis]. Three of its key features are that it (a) encourages context-sensitive health service provision by foregrounding people’s viewpoints of health priorities and solutions, (b) recognizes humans as intrinsically social beings (i.e. expanding away from individualistic models of behavior and the primacy of physical health), and (c) considers the dynamically evolving relationships between health service providers and the local population [3,35,36].

Engagement activities that emphasize relational and social aspects of healthcare challenges (and people’s agency therein) also arose during the COVID-19 pandemic [23,37]. A rapid review by Loewenson et al. [37] highlighted already 42 such cases across the globe by September 2020, spanning activities as wide-ranging as community-driven co-production of pandemic responses (e.g. social protection and food banks), the creation of inclusive spaces to articulate health needs, or support to facilitate relationships between local populations and influential stakeholders from within and outside the healthcare sector. However, and while indicative evidence has been encouraging [22,23], the overall impact evidence of community engagement activities during the COVID-19 pandemic – with its unprecedentedly systemic and global impact – remained limited and focused primarily on risk communication and knowledge provision from the top down [21,38]. Aside from the short assessment window of the pandemic, systematic evidence is also lacking because methods to evaluate the effectiveness of community engagement have developed comparatively slowly [12,39–41]. The lack of methodological guidance is especially problematic for forms of engagement that are based on fundamental social mechanisms, such as trust and on creative, symbolic, or non-verbal forms of expression [39,40,42,43], which can leave interventions unaware of their unintended positive and negative side-effects. At the same time, the practical innovativeness of relational community engagement activities such as CONNECT requires ongoing adaptation.

The extensive monitoring and evaluation work surrounding the CONNECT initiative across sectors (policy, community, healthcare) affords opportunities for the evaluation to be of broader practical relevance through an empirical, methodological, and conceptual contribution to public health evaluation knowledge [44,45].

## Material and methods

### Research design

Embedded within a larger multi-sector process evaluation of the CONNECT initiative (involving policy, health sector, and community-level assessments) [19], this study specifically examines indicators of outcomes related specifically to COVID-19 prevention and health-seeking behavior that arise from the village-based M2 activities of CONNECT. The study design compared people with different levels of participation in a quasi-experimental setting without control using mixed qualitative and quantitative methods including:

- Two rounds of complete census survey data for all available adults aged 16 years and above in 14 diverse case studies across four provinces where CONNECT activities took place.
- Complementary semi-structured interviews and qualitative survey implementation observations (field notes) alongside the village census survey for questionnaire testing and to contextualize the survey responses.
- Expert interviews with healthcare stakeholders from the local to central government levels, village representatives, and villagers to provide complementary insights into the implementation and perception of the CONNECT initiative.

**Figure 1.**
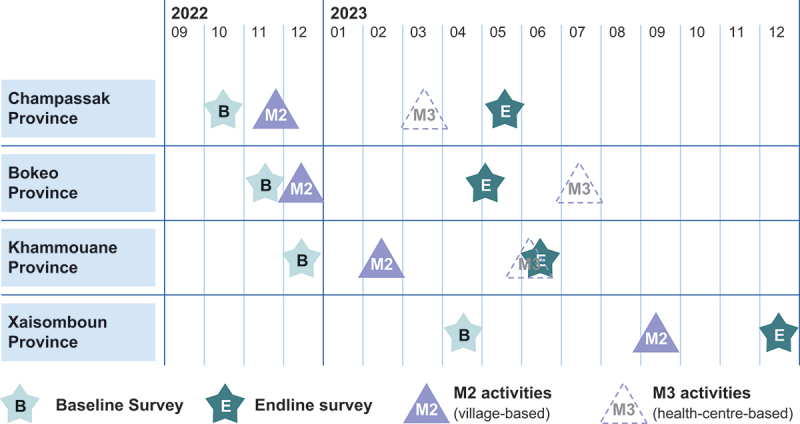
Implementation process of CONNECT village-based activities and evaluation.

The two-round census survey design permitted us to trace changes in attitudes and practices among participants and non-participants of the CONNECT activity, taking into account the complete social composition of the participating communities, and thus community-level changes. The quantitative survey data further enabled difference-in-difference (i.e. before/after) analyses among villagers with different levels of participation (i.e. participants and those aware of CONNECT but who did not participate in its activities), and non-participants as a ‘control group’ within the community setting. Qualitative methods contextualized and validated the quasi-experimental quantitative analysis results. The research was implemented between October 2022 and December 2023 (see timeline in Figure 1) by a 7-member Lao team experienced in community development and who received 5 days of mixed-method evaluation and survey training. We received ethical approval from the Lao National Ethics Committee for Health Research (NECHR; ref. 069/NECHR). Prior informed consent was obtained from all study participants.

Evaluation focuses on M2 village-based activities. M3 health-centre-based activities are shown for illustration. At the time of this manuscript, M3 activities in Xaisomboun Province were scheduled for Q2/2024.

### Research sites

**Figure 2.**
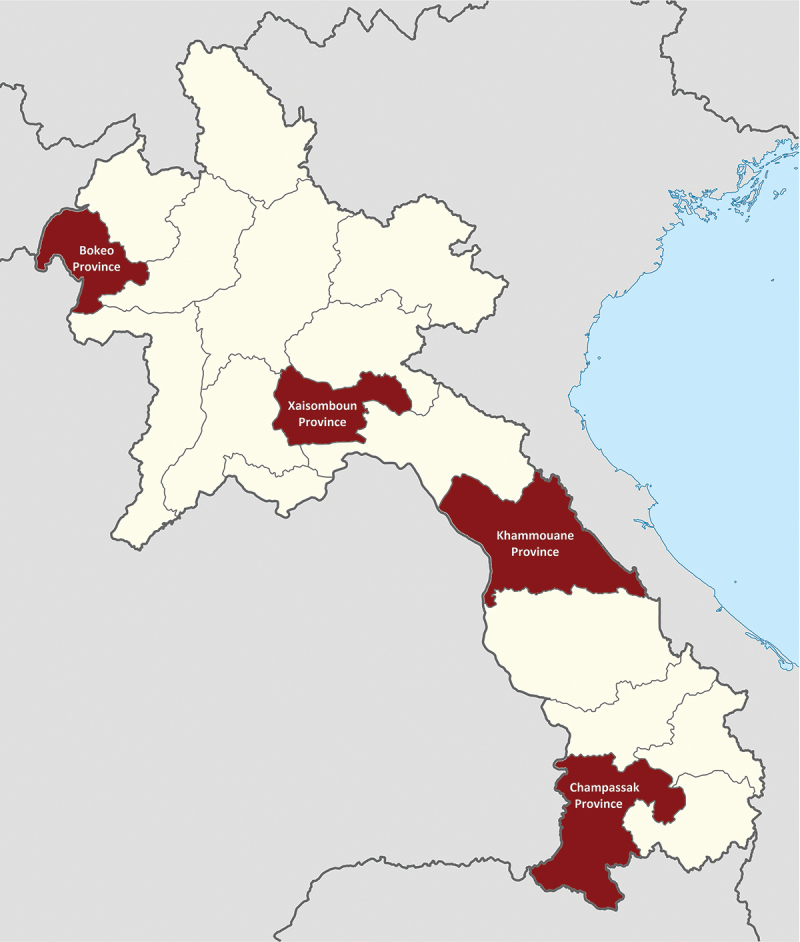
Map of Lao PDR and study sites.

**Source**: Adapted from Wikimedia Commons [48], file: Laos provinces.svg. User: Infernoapple.

**Notes**: Study provinces were highlighted in red; Bokeo province was also site for the preliminary qualitative research.

The study setting of rural Lao PDR represents a lower-middle-income country context with rapid development progress yet persistent healthcare challenges and inequalities [46]. The study sites were the provinces of Bokeo, Xaisomboun, Khammouane, and Champassak (Figure 2). Latest poverty measurement data by Coulombe et al. [47] from the year 2015 indicated that Bokeo had a poverty headcount ratio of 25.5% and an adult literacy rate of 67.0%, with corresponding values in Xaisomboun of 27.8% and 74.4%, in Khammouane of 27.1% and 83.5%, and in Champassak of 22.8% and 91.2%. All provinces together represented a selection of geographically, economically, and ethnically diverse settings. In each province, three to four communities (numbers depending on the community size and distribution) were selected as case studies. The selection of this total of 14 case study communities was purposive to capture geographical, economic, and ethnic diversity within the provinces, and all communities participated in the CONNECT initiative’s village-based activities. Following the CONNECT objectives of reaching marginalized communities who experienced challenges in maternal and child healthcare uptake, the villages were selected with varying levels of primary healthcare service uptake (prioritizing low uptake settings), remoteness, village size, and ethnic diversity. Supplementary Material Table A1 summarizes the characteristics of the study sites.

### Data collection procedures

#### Quantitative data collection

Survey data from the case study villages were collected in a two-round census survey design, wherein all available members of the 14 case study communities (aged 16 and above) were invited to participate in a baseline survey and a follow-up survey at least 3 months after the first CONNECT activities (workshops and subsequent supportive supervision activities did not always coincide and participants’ attendance had been recorded separately) – emulating the survey data collection process described in Haenssgen et al. [49]. The 40-min questionnaire covered several modules to evaluate the village-based activities, including respondent/household characteristics (baseline), CONNECT activity participation (endline), and trust and health system relationships (base-/endline). Specific to the evaluation of COVID-19-related outcomes were modules focusing on COVID-19 attitudes and practices (base-/endline; information-seeking behavior, vaccination readiness, stigma) and healthcare utilization (base-/endline; patterns of healthcare utilization for acute illness with a 2-month recall window).

The questionnaire was developed in Lao with the help of the survey team experienced in community development projects. The survey data collection used an interviewer-administered questionnaire operating on tablets using *SurveyCTO* [50]. A survey pilot took place in Champassak Province and was followed by a further round of expert review, which entailed minor revisions of the questionnaire (the data remained comparable across all provinces).

#### Qualitative data collection

Aside from qualitative survey implementation notes collected by the survey team, the qualitative material had two main sources [51]. Firstly, semi-structured 20–30-min interviews accompanied the survey to interpret and contextualize the survey findings, involving community members and various community-based authorities and stakeholders (e.g. village chiefs and village health volunteers in their role as community members). The semi-structured interview guide followed the main modules and key questions of the questionnaire. Secondly, 30–45-min semi-structured key informant interviews with community members and representatives, village authorities, health center staff, as well as CONNECT stakeholders on the district, provincial, and central levels helped provide further information related to the policy context and higher-level implementation of the CONNECT initiative. The key informant interview guide followed the evaluation criteria that guided the CONNECT evaluation (efficiency, effectiveness, relevance, coherence, impact, sustainability, and equity; based on [41] and [52]). Interview participants were recruited purposively to reflect the diversity of communities and provinces and to include views among all stakeholder levels.

Similar to the survey questionnaire, the interview guides were developed in Lao with the help of the survey team. The interview data were collected through face-to-face interviews, which were audio-recorded, transcribed, and translated into English by the evaluation team. All survey and qualitative data were collected in Lao or in the preferred ethnic languages of the respondents (for which we recruited local translators), and participants received a small in-kind gift as a token of appreciation for their time and participation.

### Data management and analysis

The resulting material (summarized in Table 2) comprised 3,161 survey observations (1,838 at baseline and 1,323 at endline, whereby endline data collection was complicated by seasonal working and migration patterns), of whom 53.0% were female and whose main ethnic groups included Lao Loum (35.4%), Hmong (20.1%), and Khmu (18.3%). In addition, 618 respondents could be followed up and matched across the two survey rounds, which enabled us to create 2-round panel data sets from a sub-sample of the survey. The qualitative data amounted to 54:02 h of audio-recorded material from 50 semi-structured interviews and 50 key informant interviews. 48.0% of all qualitative participants were female and the subset of interviews involving villagers and village authorities (71 out 100 interviews) included a relative majority of 28.2% lowland Lao (Lao Loum) ethnicity, Hmong (25.4%), and Khmu (19.7%) among others. The qualitative and quantitative data thus over-represented ethnic minority groups (in line with the intentions of CONNECT and the evaluation) as per the latest 2015 census ‘Out of the total population, the Lao ethnic group accounted for 53%, followed by Khamu (11%), Hmong (9%)’ [53].

**Table 2.** Overview of village-based evaluation data.

	Province					
	Champassak	Bokeo	Khammouane	Xaisomboun	Central level	Total
**Interviews**	**20**	**23**	**23**	**30**	**4**	**100**
*Semi-structured Interviews*	11	11	11	17		50
Baseline	*5*	*4*	*7*	*10*		*26*
Endline	*6*	*7*	*4*	*7*		*24*
*Key Informant Interviews*	9	12	12	13	4	50
Baseline	*3*	*5*	*5*	*4*	*4*	*21*
Endline	*6*	*7*	*7*	*9*		*29*
**Survey observations**	**1,005**	**835**	**354**	**518**		**3,161**
Baseline	658	440	222	518		1,838
Endline	347	395	132	449		1,323

These data were managed centrally by WHO Lao PDR. The project principal investigator was responsible for overseeing data management as well as the development and execution of planned analyses. Qualitative transcripts and handwritten notes were de-identified at source and integrated into the qualitative data analysis software MAXQDA 2020 [54] in digital format; paper records of notes were subsequently shredded. Survey data were collected in anonymous form and stored on a secure 2048-bit SSL encrypted server operated by SurveyCTO [50], from where the data were downloaded and further analysis using Stata 16 [55]. All qualitative and quantitative analytical data files were stored in an access-restricted password-protected online drive maintained by WHO Lao PDR, and any external storage devices containing electronic data were overwritten and deleted by an irrecoverable process.

Following a two-step analysis strategy, we first analyzed the qualitative material through content-oriented thematic analysis to understand and contextualize the implementation setting [51]. Using MAXQDA 2020, the analysis identified and coded any evaluation-related content and subsequently categorized it into patterns (themes), whereby the diversity of respondents and settings helped to actively source negative cases to challenge evolving findings and to provide nuance for the assessment [51]. The subsequent quantitative analysis aimed at ascertaining the outcomes of the CONNECT activities systematically and at scale through descriptive statistical analysis (non-inferential due to the use of census data) [56]. The two-round design made it possible to assess indicator changes in the case study communities before and after the CONNECT activities and among individuals who took part in, were merely aware of (e.g. via word-of-mouth), and who remained unaware of the activities. We further analyzed the panel data set comprising 618 individuals in a difference-in-difference framework.

Note that in both the qualitative and quantitative analysis, we were conscious of potential issues of social desirability biases of participants responding intentionally positively about their participation in the CONNECT initiative. We mitigated this issue by focusing less on people’s personal evaluation of the CONNECT activities (unless where relevant as stakeholder) and rather on behavioral outcomes such as received vaccine doses or health behavior (which respondents were not asked to directly link to the CONNECT activities). For example, the sequential data on treatment-seeking experiences elicited step-by-step people’s medicine use, which did not only aid the recall of healthcare episodes [57,58] but also led respondents to focus their attention specifically on the detailed healthcare experiences rather than their previous participation in the CONNECT activities.

## Results

### Baseline context of COVID-19

At the time of the baseline data collection (October 2022 to April 2023), the situation in the case study communities was characterized by ongoing challenges in attaining comprehensive vaccination coverage. COVID-19 vaccination and related stigma featured regularly in the qualitative accounts from healthcare stakeholder (e.g. interviews in Khammouane, Provincial Health Office, Baseline; Bokeo, District Health Office, Baseline), and health center staff serving a Bokeo village lamented for instance the difficulties of ‘*giving the COVID vaccination shots to villagers because some of them didn’t go* [to the health center] *and get the vaccine as they should*’ (Bokeo, Health Center, Baseline). In Xaisomboun, health center staff elaborated further on the reasons for the lacking vaccination coverage:

1. *The villagers still lack perception about the importance of information.*
2. *Some of the staff are still not able to spread the information and knowledge to the villagers.*
3. *Villagers would say that, ‘We don’t need to get vaccinations. When we get sick, we will go to the health center and the staff will treat us anyways.’*
4. *Sometimes when we visited the villagers’ houses, they would not be there although we had sent them a letter about our visit.*
5. *Some villages didn’t cooperate and that created inconsistent data.*

(Xaisomboun, Health Center, Baseline)

Villagers themselves corroborated some of these challenges. From their perspective, the information landscape surrounding COVID-19 was scattered and inconsistent. Only 13.2% of all the villagers in the survey would obtain information about disease outbreaks from public healthcare sources, while the five other top sources mentioned by 93.4% of the villagers indicated a high reliance on announcements from village authorities (52.8%), TV or radio information (44.8%), other villagers (31.4%), social media (31.2%), or family members (9.2%). That the quality of COVID-19-related information from these sources can be questionable was illustrated by a villager in Bokeo Province, who reported that their Village Chief announced to all villagers to eat boiled eggs in order to be safe from the virus – and consequently an egg shortage ensued (survey observations, Bokeo, Baseline).

While many villagers remained unconcerned about COVID-19, the disease was still occasionally associated with fear and stigma. A villager in Champassak relayed in this context how she experienced stigma as she contracted COVID-19: other people around her would gossip that, ‘*On that day, I saw this person* [i.e. the respondent] *come to the market, and after that that market had been closed immediately*’ (Champassak, Villager, Baseline). Market closures in response to outbreaks were of course nothing unusual, but the experience of the respondent to be actively singled out and pointed at by other villagers had created an emotional burden. Another villager in the same province reported that she had received two vaccine doses but became ill with COVID-19 with a traumatizing social impact for her:

*I went to the rehabilitation center* [after getting COVID-19]. *My cousins did not want to get close to me, they were disgusted by me. No one ever asked me how I was, not even the chief of the village. No one! They were all afraid of me. They were afraid to die.*

(Champassak, Villager, Baseline)

Yet, few villagers were aware of anyone ever having contracted COVID-19 (13.9% on average). At the same time, the survey respondents indicated that they received 2.23 COVID-19 vaccine doses while considering 3.13 to be the optimal number – provided they were confident in articulating such a figure (38.0% answered ‘don’t know’). While variation in vaccine coverage across socio-economic strata was relatively uniform (ranging from 2.10 to 2.41 received doses and 2.91 to 3.45 optimal vaccine doses), the province case studies exhibited in Bokeo a positive outlier with 2.90 received and 3.97 optimal vaccine doses (the remaining three provinces had uniform rates that were accordingly slightly below average). However, still 9.6% remained unvaccinated at baseline (common reasons included absence during the campaigns ranked with [16.2%], existing illnesses at the time of the campaigns [16.2%], being afraid of the vaccine [15.0%], pregnancy [14.5%], or preferring not to get vaccinated [9.8%]).

Although 23.1% of the baseline participants reported an illness or injury over the 2 months preceding the survey (11.8% if only adult respondents commenting on themselves are included), no participant explicitly reported COVID-19 as an actual or suspected diagnosis (see Supplementary Material Figure A1 for a breakdown of reported illnesses by presentation). However, 3.0% of the respondents reported fever symptoms and an additional 4.4% fever with neurological and/or respiratory presentation that were consistent with common COVID-19 symptoms (respiratory presentation alone was reported in 7.0% of cases). This is plausible in principle as Lao PDR rarely registered more than 1000 cases per week between August 2022 and March 2023 (most of which were concentrated in Vientiane Capital) and 80.1% of the population had received at least one dose of the COVID-19 vaccine by 31 July 2022, while under-reporting could be ruled out as only 500–1000 tests were conducted daily and test positivity often exceeded 20% [59]. The healthcare access and utilization patterns associated with the different presentations are shown in Table 3 and suggest that febrile patients had relatively high public healthcare access, while additional symptoms were linked to higher private and informal healthcare access as well (note that CONNECT aimed primarily at public healthcare utilization as it built trust between communities and their public healthcare providers alongside skill development activities at public primary care facilities; private healthcare access through clinics requires higher out-of-pocket expenditure but is not necessarily inferior to public healthcare access; however, informal healthcare access including retired practitioners or faith healers can create concerns about the quality of care for serious health conditions). Patients presenting with fever and neurological and/or respiratory symptoms also had with 0.37 antibiotic use episodes per illness episode the highest average antibiotic consumption among the different presentation categories. This indicates that patients with COVID-19-consistent symptoms may have been at increased risk of injudicious antibiotic use.

**Table 3.** Healthcare access and utilization indicators at baseline.

Presentation	Healthcare access			Antibiotic use episodes
	Public	Private	Informal	
Fever only	73.1%	5.8%	11.5%	0.21
Fever plus neurological and/or respiratory	51.5%	8.8%	30.9%	0.37
Respiratory only	33.7%	16.3%	19.8%	0.35
Other presentation	66.1%	3.5%	9.6%	0.20
**Average**	**55.5%**	**8.4%**	**17.1%**	**0.28**

Baseline data, based on respondents who reported a completed injury or illness episode in the two months preceding the survey either for themselves or a child under their supervision (*N* = 321). Presentation categories are mutually exclusive; multiple forms of healthcare access possible within one illness episode.

### CONNECT workshop implementation

Overall, 283 out of 1,323 respondents (21.4%) in the endline sample attended either the CONNECT M2 workshop or the subsequent supportive supervision activities, plus a further 100 (7.6%) who attended both events at most partially. In other words, a substantial fraction of the case study village population participated in the village-based CONNECT activities, and virtually everyone who was invited to the activities attended at least one of them (30.2% invited vs. 29.0% who attended at least one event at least partially). Among the participants, approximately three-quarters felt involved in either case of the M2 workshops or the supportive supervision activities. In addition, 332 respondents (36.9%) were aware that village-based CONNECT activities took place, meaning that 105 individuals or 7.9% gained exposure to the activities indirectly via village authorities and other community members.

**Figure 3.**
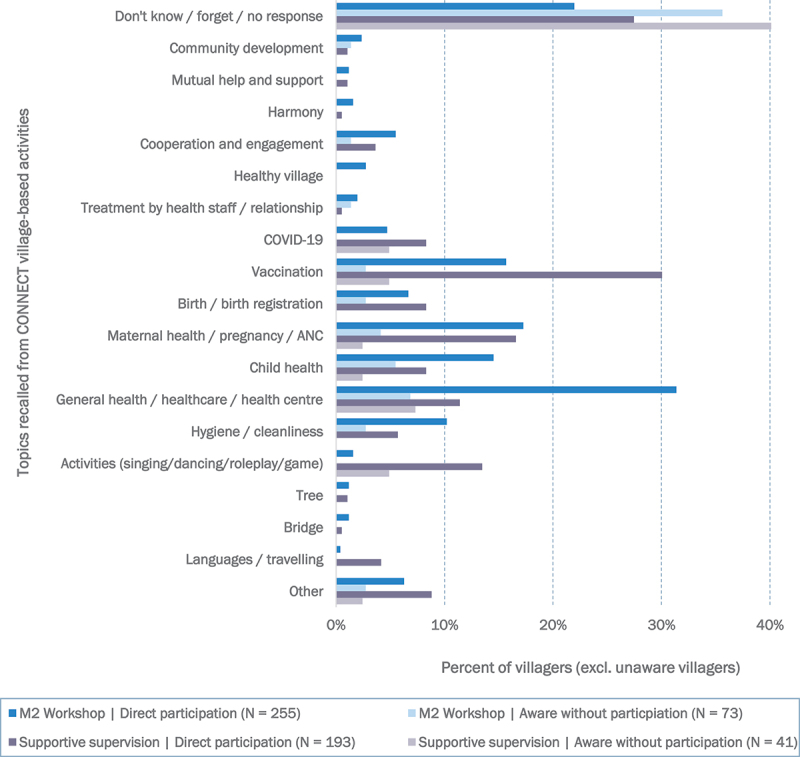
Interpretations of CONNECT M2 workshops and supportive supervision by level of participation (direct participation vs. being aware without participation).Percentage share based on respective subgroup. Multiple responses possible; coded based on free-text responses

Among the participants, the absorption and retention of the activity content was mixed (Figure 3). Approximately one-quarter of the workshop and supportive supervision participants could not recall any topics from the activities (22% and 27%, respectively), and only 4.7% of the workshop participants and 8.3% of the supportive supervision participants associated the activities with COVID-19, while 15.7% and 30.1%, respectively, recognized the broader theme of vaccination (including COVID-19 but also maternal and childhood vaccines). Topics relating to general health and maternal and child healthcare found broader recognition among the participants, but COVID-19-related content was relatively poorly understood among non-participants who learned about CONNECT through word-of-mouth (light-shaded bars in Figure 2). In addition, villagers were in many instances unable to articulate the content or objectives of the activities at all, referring in many instances to the nature (rather than content) of the activities or simply explaining that they did not understand the activities and ‘*did not learn anything. Just sit and watch*’ (Bokeo, Villager, Endline). Villagers who were only aware of the activities (without participation) were yet more likely to simply state that they did not know what the CONNECT activities were about whilst also the retention of the activities was limited as villagers ‘*could understand at that time, but now I forgot it*’ (Champassak, Villager, Endline). Even village authorities would observe this as an issue as they stated that, ‘We have explained [about the CONNECT activities] *in the past and people may have remembered, but now they have completely forgotten*’ (Khammouane, Village Head, Endline).

### Intervention effects on COVID-19 indicators

The effects of the CONNECT activities were positive overall but also pointed at potentially challenging developments. Qualitative descriptions of COVID-19-related activity outcomes revolved primarily around vaccination. For instance, on the provincial level, a member of the Public Health Office in Xaisomboun Province highlighted that the improved coordination between their team, the Office of Home Affairs, and the Security Office when helping to ‘*encourage COVID-19 vaccinations*’ (Xaisomboun, Provincial Health Office, Baseline [respondent had previously participated in CONNECT activities]), and a respondent on the district level similarly emphasized that, ‘*after CONNECT comes in, we explain that people will have to get the COVID vaccine, that nobody needs to be afraid because everyone gets the vaccine to reduce the risk*’ (Champassak, District Health Office, Endline).

On the community level, health center staff and village authorities also stressed the effects on COVID vaccine uptake and their work in reducing people’s fear surrounding vaccine side-effects. The improvements were tangible from the perspective of the respondents, who expressed that ‘*the number of COVID vaccines is increasing as well as child vaccination*’ (Champassak, Health Center, Endline), that ‘*people didn’t get* [the COVID vaccine] *at first because they were afraid, but after they know about the information* [from CONNECT], *they come and get the vaccine*’ (Champassak, Village Health Volunteer, Endline), and that, ‘*after CONNECT happened, when we explained to the people, they understood faster. It was not as difficult as before that we have to go and find them when we went for immunization*’ (Khammouane, Village Chief, Endline). Villagers themselves corroborated these impressions. Attendees of the activities recalled that, ‘*They divided us into groups. They did a performance about COVID and vaccines*,’ but they also directly observed the effects of such activities: ‘*after watching their performance, some villagers were interested in the vaccines and took their kids to get vaccinated after*’ (Khammouane, Villager, Endline). Elsewhere, villagers would describe the changes after the CONNECT activities as, ‘*before that* [workshop], *the guy near my house did not take a vaccine, but after they came, he has already taken 2–3 shots by now*’ (Champassak, Villager, Endline).

The statistical data support these observations (summarized in Table 4): Firstly, although the share of people receiving COVID-related information from the health center dropped from its low level across the survey rounds, villagers participating in the CONNECT activities were at least 6.6%-points more likely to report the health center as an information source compared to villagers who were not aware of CONNECT. Secondly, the average number of received COVID vaccines before the workshop was 2.24, while villagers reported on average 2.61 vaccines at the endline (i.e. approximately 0.4 doses higher) – whereby participants of the CONNECT activities (2.86) and those aware of it (2.84) had an approximately 15.4% higher vaccination count than villagers who were unaware of the CONNECT activities (2.46). While this is encouraging, the perceived optimum number of vaccine shots was in fact 0.47 doses *lower* in the endline survey (primarily as responses shifted from ‘don’t know’ to zero doses). At the same time, villagers participating in CONNECT had above-average expectations about the number of optimal vaccine doses, which suggests a positive influence of the activities amidst generally waning interest in getting vaccinated (note that information about perceived optimal doses was gathered not to test [contested] factual knowledge but to elicit personal perception on the balance between received and envisaged vaccine doses). However, participation in the workshop and the ensuing (re-)sensitization to COVID-19 could potentially also create a counter-reaction as the CONNECT participants were on average more worried (49.9%) than their unaware counterparts (40.9%) to disclose a COVID-positive status. Lastly, overall patterns of healthcare utilization also speak in favor of the CONNECT activities as general public healthcare utilization during illness was consistently higher, and antibiotic use consistently lower, among the survey respondents at endline and activity participants.

**Table 4.** Key COVID-19-related outcome indicators across survey rounds and by endline participation.

	Health center as source of COVID information	Received no. of COVID vaccine doses	Perceived optimal COVID vaccine doses		Worried to disclose positive COVID status	Healthcare utilization^a^		*N*
			No.	% don’t know		Public healthcare	Antibio-tic use episodes	
**By survey round**								
Baseline	10.1%	2.24	3.14	38.0%	40.5%	55.6%	0.28	1,838
Endline	8.7%	2.61	2.67	19.1%	43.1%	62.1%	0.15	1,323
**By participation in CONNECT village-based activities at endline**								
Unaware of CONNECT	6.2%	2.46	2.46	18.0%	40.9%	56.5%	0.24	835
Aware, no participation	13.3%	2.84	3.28	28.6%	35.6%	59.1%	0.18	105
Participated	12.8%	2.86	2.98	19.1%	49.9%	67.1%	0.21	383

^a^Subsample of respondents with completed illness episodes in respective survey round, *N* = 322 at baseline and *N* = 95 at endline (with 54, 11, and 30 observations, respectively, for unaware, aware, and participating participants).

More nuanced insights into the short-term impacts of CONNECT can be gleaned from the subsample of respondents who were followed up in the endline survey. Presented in detail in Table 5, a key observation is the tendency of villagers with more favorable COVID-19-related indicators at baseline to self-select into joining the CONNECT activities – which is unsurprising as the community-based activities are unlikely to recruit a random cross-section of the local population. For example, while activity participants were at the endline most likely to recommend health centers specifically as a treatment option for COVID-19 (59.6% of the panel) and unaware villagers were least likely (54.6%), the latter exhibited a noticeably larger increment of 16.4% points from its low base of 38.2% (vs. 10.1 p.p. for participating villagers). Nevertheless, the overall effects of CONNECT remain favorable when considering the difference-in-difference between the changes among CONNECT participants and unaware individuals. For instance, participating villagers were relatively more likely to consider the health center as a source of COVID-19 information, had at least relatively higher actual and perceived optimal numbers of COVID-19 vaccine doses, and exhibited absolutely and relatively higher public healthcare utilization for COVID-19-related symptoms. From a global health perspective, especially noteworthy is also the absolutely and relatively reduced antibiotic usage for COVID-19-related symptoms among CONNECT participants. However, among other illness presentations that did not include COVID-19 symptoms, participating villagers exhibited relatively lower public and higher informal healthcare utilization at the endline, and the participants appeared to become worried toward disclosing a COVID-positive status. While space constraints do not permit an exhaustive analysis by province and case study village, indicative explorations suggest that the positive trend of the outcome indicators persists across provinces as, for example, all provinces exhibited relatively higher or at least non-negative changes of actual and perceived optimal numbers of COVID-19 vaccine doses (ranging from +0.0005 to +0.46 and +0.24 to +1.04, respectively) between CONNECT participants and unaware villagers.

**Table 5.** Key COVID-19-related outcome indicators in matched panel dataset.

Indicator			Level of participation in CONNECT village-based activities								First difference				Difference-in-difference		N
			Unaware		Aware		Participated		Average						Participated vs. unaware	Aware vs. unaware	
			Baseline	Endline	Baseline	Endline	Baseline	Endline	Baseline	Endline	Unaware	Aware	Participated	Average			
Recommending health center for COVID care			38.2%	54.6%	40.4%	55.8%	49.5%	59.6%	42.4%	56.5%	+16.4 p.p.	+15.4 p.p.	+10.1 p.p.	+14.1 p.p.	−6.3 p.p.	−1.0 p.p.	1236
Recommending any public healthcare for COVID care			65.2%	81.6%	75.0%	88.5%	76.6%	82.6%	70.1%	82.5%	+16.4 p.p.	+13.5 p.p.	+6.0 p.p.	+12.5 p.p.	−10.4 p.p.	−2.9 p.p.	1236
Health center as source of COVID information			11.2%	6.3%	19.2%	13.5%	16.1%	13.8%	13.6%	9.5%	−4.9 p.p.	−5.8 p.p.	−2.3 p.p.	−4.0 p.p.	+2.6 p.p.	−0.9 p.p.	1236
Received doses of COVID-19 vaccine^a^			2.22	2.56	2.41	3.02	2.53	3.00	2.34	2.76	+0.34	+0.61	+0.48	+0.41	+0.13	+0.26	1234
Optimal number of COVID-19 vaccine^a^			3.18	2.27	3.46	3.41	3.40	2.97	3.29	2.61	−0.91	−0.05	−0.43	−0.68	+0.48	+0.86	878
Worried to disclose COVID-19 positive status^a^			43.9%	41.2%	36.7%	38.8%	36.2%	48.8%	40.6%	43.8%	−2.7 p.p.	+2.0 p.p.	+12.6 p.p.	+3.1 p.p.	+15.3 p.p.	+4.7 p.p.	1187
Healthcare utilization^b^	Public	Fever only	87.5%	41.7%	100.0%	100.0%	75.0%	71.4%	81.0%	55.0%	−45.8 p.p.	0.0 p.p.	−3.6 p.p.	−26.0 p.p.	+42.3 p.p.	+45.8 p.p.	41
		Fever + neurol. and/or respir. presentation	44.4%	0.0%	40.0%	100.0%	75.0%	100.0%	48.1%	75.0%	−44.4 p.p.	+60.0 p.p.	+25.0 p.p.	+26.9 p.p.	+69.4 p.p.	+104.4 p.p.	31
		Respiratory presentation only	40.0%	50.0%	100.0%	100.0%	38.5%	100.0%	41.7%	70.0%	+10.0 p.p.	0.0 p.p.	+61.5 p.p.	+28.3 p.p.	+51.5 p.p.	−10.0 p.p.	34
		Other presentation	60.0%	66.7%	50.0%	100.0%	81.8%	83.3%	65.0%	81.8%	+6.7 p.p.	+50.0 p.p.	+1.5 p.p.	+16.8 p.p.	−5.2 p.p.	+43.3 p.p.	51
		Average	55.7%	45.5%	54.5%	100.0%	65.0%	83.3%	58.9%	66.7%	−10.3 p.p.	+45.5 p.p.	+18.3 p.p.	+7.7 p.p.	+28.6 p.p.	+55.7 p.p.	157
	Private	Fever only	0.0%	8.3%	0.0%	0.0%	8.3%	0.0%	4.8%	5.0%	+8.3 p.p.	0.0 p.p.	−8.3 p.p.	+0.2 p.p.	−16.7 p.p.	−8.3 p.p.	41
		Fever + neurol. and/or respir. presentation	5.6%	100.0%	20.0%	0.0%	50.0%	0.0%	14.8%	25.0%	+94.4 p.p.	−20.0 p.p.	−50.0 p.p.	+10.2 p.p.	−144.4 p.p.	−114.4 p.p.	31
		Respiratory presentation only	10.0%	0.0%	0.0%	0.0%	30.8%	0.0%	20.8%	0.0%	−10.0 p.p.	0.0 p.p.	−30.8 p.p.	−20.8 p.p.	−20.8 p.p.	+10.0 p.p.	34
		Other presentation	0.0%	33.3%	0.0%	0.0%	0.0%	0.0%	0.0%	9.1%	+33.3 p.p.	0.0 p.p.	0.0 p.p.	+9.1 p.p.	−33.3 p.p.	−33.3 p.p.	51
		Average	3.3%	13.6%	9.1%	0.0%	17.5%	0.0%	8.9%	6.7%	+10.4 p.p.	−9.1 p.p.	−17.5 p.p.	−2.3 p.p.	−27.9 p.p.	−19.4 p.p.	157
	Informal	Fever only	0.0%	8.3%	0.0%	0.0%	8.3%	0.0%	4.8%	5.0%	+8.3 p.p.	0.0 p.p.	−8.3 p.p.	+0.2 p.p.	−16.7 p.p.	−8.3 p.p.	41
		Fever + neurol. and/or respir. presentation	33.3%	0.0%	20.0%	0.0%	25.0%	0.0%	29.6%	0.0%	−33.3 p.p.	−20.0 p.p.	−25.0 p.p.	−29.6 p.p.	+8.3 p.p.	+13.3 p.p.	31
		Respiratory presentation only	10.0%	16.7%	0.0%	0.0%	7.7%	0.0%	8.3%	10.0%	+6.7 p.p.	0.0 p.p.	−7.7 p.p.	+1.7 p.p.	−14.4 p.p.	−6.7 p.p.	34
		Other presentation	20.0%	0.0%	25.0%	0.0%	9.1%	16.7%	17.5%	9.1%	−20.0 p.p.	−25.0 p.p.	+7.6 p.p.	−8.4 p.p.	+27.6 p.p.	−5.0 p.p.	51
		Average	19.7%	9.1%	18.2%	0.0%	10.0%	5.6%	16.1%	6.7%	−10.6 p.p.	−18.2 p.p.	−4.4 p.p.	−9.4 p.p.	+6.1 p.p.	−7.6 p.p.	157
Antibiotic use episodes^b^		Fever only	0.13	0.17	1.00	0.00	0.08	0.00	0.14	0.10	+0.04	−1.00	−0.08	−0.04	−0.13	−1.04	41
		Fever + neurol. and/or respir. presentation	0.50	1.00	0.00	0.00	0.50	0.50	0.41	0.50	+0.50	+0.00	+0.00	+0.09	−0.50	−0.50	31
		Respiratory presentation only	0.20	0.17	0.00	0.00	0.62	0.00	0.42	0.10	−0.03	+0.00	−0.62	−0.32	−0.58	+0.03	34
		Other presentation	0.20	0.00	0.25	0.50	0.27	0.00	0.23	0.09	−0.20	+0.25	−0.27	−0.13	−0.07	+0.45	51
		Average	0.28	0.18	0.18	0.20	0.35	0.06	0.29	0.13	−0.10	+0.02	−0.29	−0.16	−0.20	+0.12	157

^a^Sample excludes ‘don’t know’ responses.

^b^Sample only includes completed illness episodes.

## Discussion

Despite their growing popularity in the global health discourse, the knowledge about relational community engagement activities to address emerging infectious disease challenges such as the COVID-19 pandemic remains circumstantial. Our study provided rare mixed-method evidence about the implementation and short-term effects of the CONNECT initiative in Lao PDR. Studying 14 diverse communities in four provinces spread across the country, our analysis demonstrated a plausible positive short-term impact on COVID-19-related attitudes and practices. We documented that CONNECT responded to pressing local healthcare needs that were partly defined and partly aggravated by the COVID-19 pandemic, and stakeholders perceived both the implementation and the outcomes of the initiative as favorable. Challenges materialized as well: Inclusion in the activities and the absorption of their content were mixed, and participants often interpreted the activities as common health education campaigns. The community embeddedness of CONNECT further tended to mobilize villagers with already more favorable COVID-19-related attitudes and practices at baseline. Finally, the interest in the pandemic gradually waned in the evolving study context, which impacted CONNECT’s ability to influence COVID-19-related attitudes and practices.

Despite these challenges, participation in the CONNECT activities was associated with:

- Increased reported numbers of COVID-19 vaccine doses and a relatively slower declining trend of perceived optimum vaccine dose numbers (which remained above the average actual number of doses).
- Heightened worry to disclose a positive COVID-19 status, likely as a result of re-sensitization during the CONNECT activities.
- An uptake of public healthcare for presentations consistent with COVID-19 (less favorable patterns inclined toward informal care materialized for other illness presentations that did not include COVID-19 symptoms).
- Lower average antibiotic use (especially for presentations consistent with COVID-19), likely by means of directing healthcare utilization toward more regulated public healthcare provision – alongside interpersonal elements of trust and respectful care that were similarly emphasized during the workshop and which may alleviate pressures of ‘quick fix’ antibiotic provision [60].

In addition, no discernible impact was found on COVID-19 information-seeking or recommended resorts to care, which either trended toward parity or otherwise showed no difference in relative movement between CONNECT participants and unaware villagers. Our study thereby substantiates the literature, indicating the positive potential of relational community engagement activities to support people’s healthcare challenges [23,37]. While for instance stigmatization is a common co-occurrence of new or poorly understood diseases, the building of trust and mutual understanding of people’s realities has been a key mechanism of the village-based CONNECT activities that distinguish the Initiative from conventional public health promotion campaigns which tend to impose desired behaviors from the top-down (see [33,34] on evaluation findings on the positive trust outcomes of CONNECT). Our study also provides nuanced insights on the implementation dynamics (e.g. re-interpretation of the CONNECT initiative), complex distribution (e.g. patterns of inclusion and exclusion in activities), and potential unintended consequences of community engagement – both positive (e.g. on reducing the risk of injudicious antibiotic use) and negative (e.g. increased sensitization toward negative social repercussions of COVID-19 status).

Despite its advantages, the nature of the mixed-method evaluation also imposes certain limitations on the conclusions of this paper. Firstly, we aided recall especially for treatment-seeking experiences by eliciting the sequence of events during recent illnesses [57,58], but the possibility of recall bias cannot be ruled out entirely and may contribute to a slight under-representation of lower-educated population groups [61]. Secondly, the in-depth study of 14 communities contains a diverse sample of cases across rural settings of Lao PDR, but it cannot claim representativeness for the country as a whole (given e.g. the over-representation of ethnic minority groups). This is linked to the third point, in that the research design mobilized in this study (as part of a broader multi-sectoral evaluation) to inform the ongoing implementation of CONNECT also limits the insights into systemic impacts affecting a community as a whole. For instance, owing to the absence of ‘control’ communities, potential spillover effects between participants and non-participants groups in the same communities cannot be entirely ruled out and need to be acknowledged (however, the geographical spread of the case study sites made cross-provincial contamination effects within this study unlikely). Also, conclusions to CONNECT’s medium- and long-term impact remain limited and indicative at this point (see e.g. indication on medium-term impacts relating to the limited retention of the activity content). Subsequent research can complement the empirical findings of this study, for instance in a quasi-experimental research design using multi-round nationally representative household survey data such as the Lao Social Indicator Survey [62].

## Conclusion

While having a long and established intellectual history, relational community engagement presents a key evolution in global health practice. Our evaluation of the CONNECT initiative in Lao PDR has demonstrated its ability to support communities in addressing local healthcare challenges related to COVID-19 with potentially beneficial side-effects on reduced antibiotic consumption for patients who presented with COVID-19-compatible symptoms – which is an important outcome as the Initiative did not actively target antibiotic resistance. Our mixed-method approach was further able to detect potentially negative unintended consequences from the sensitization of target populations and unravel the distributional patterns of the short-term impact on COVID-19-related attitudes and practices. While such implementation dynamics can be expected in any new policy initiative [63], open-ended exploratory research designs allow program managers to subsequently address these issues through operational adjustments. Overall, these results speak in favor of CONNECT’s ability to support Lao national health policy priorities as the country transitions from the COVID-19 pandemic into a new phase of macroeconomic and geopolitical challenges [15,17].

To contribute to the ongoing development of CONNECT and other relational community engagement initiatives, further means to espouse bottom-up feedback from the communities to health centers and higher-level stakeholders could help sensitize stakeholders toward the lived experiences (such as stigma) of the local populations and their realities of exclusion and inequity. Longer-term implementation of the initiative with local co-ownership among communities and healthcare providers can also help to direct the Initiative’s focus stronger toward relational aspects of care as opposed to mere health education, while also providing opportunities to include hitherto unreached segments of the community (especially groups with poor health status or other livelihood constraints that prevented inclusion in the CONNECT activities). CONNECT’s adaptive implementation approach thereby offers scope for a more extensive content translation – not only to reach more non-Lao-speaking population groups but also conceptually to relate the activity content closer to the lived experiences of the target communities. Owing to the long-term multi-stakeholder development process of CONNECT, health departments and primary healthcare units have expressed their readiness for implementing CONNECT-style community engagement activities at scale, but resource-limited contexts such as Lao PDR also require more extensive financial and human resource support to realize such developments on the primary healthcare level [15].

More generally, the encouraging findings of the evaluation support the rationale of grounded community engagement initiatives to become a standard component of national health policy portfolios – beyond the scope of COVID-19 [3,12,64,65]. However, this process ought to avoid the risk of losing the spirit of engagement and reducing the initiative to the relatively common interactive health education or community mobilization campaigns [1,21,24]. In the case of CONNECT, key mechanisms to retain this spirit were especially the grounded nature of the initiative (driven by issues experienced on the community level and supported by local influential persons), a positive engagement approach that was free of blame and focused on mutual support, stakeholder buy-in on all levels from the community to the central level, and inter-institutional coordination between different government agencies. Furthermore, our evaluation underlined the potential of community-based engagement activities to address the social complexities of injudicious antibiotic use and thus contribute to tackling a pressing global health priority through socially grounded mechanisms [60,66–70].

## Data Availability

The datasets generated and analyzed during the current study are not publicly available to protect the anonymity of our respondents but are available from the corresponding author on reasonable request.
